# Sex Differences in Admission Urine Culture Positivity, Pathogen Distribution, and Clinical Characteristics Among Patients with Calcium Oxalate Stones

**DOI:** 10.3390/pathogens15070692

**Published:** 2026-06-30

**Authors:** Xijie Ding, Jianxing Li, Guojun Chen, Chaoyue Ji, Weiguo Hu

**Affiliations:** 1 Department of Urology, Qinghai University Affiliated Hospital, School of Clinical Medicine, Qinghai University, Xining 810000, China; 2 Department of Urology, Beijing Tsinghua Changgung Hospital, School of Clinical Medicine, Tsinghua Medicine, Tsinghua University, Beijing 102218, China

**Keywords:** calcium oxalate stones, urine culture, sex, urinary biochemical parameters, kidney stones

## Abstract

**Purpose**: This study aimed to determine whether positive admission urine culture is associated with stone burden, renal involvement, pathogen distribution, and measured urinary biochemical profiles in men and women with calcium oxalate stones. **Methods**: We retrospectively analyzed adults who underwent percutaneous nephrolithotomy or ureterorenoscopy for upper urinary tract stones between 2016 and 2020. Calcium oxalate stones were defined as stones containing ≥50% calcium oxalate monohydrate and/or dihydrate by Fourier transform infrared spectroscopy. Patients were compared by sex and then stratified by admission urine culture status within each sex. **Results**: Among 1257 patients, 878 were men and 379 were women. Women had a higher culture-positive rate than men (59.6% vs. 28.2%, *p* < 0.001), despite lower 24-h urinary calcium, uric acid, sodium, potassium, phosphorus, and chloride. In men, culture positivity was associated with recurrent stones, renal stone location, larger maximum stone diameter, and lower eGFR, but not with measured 24-h urinary parameters. In women, culture positivity was associated with renal stone location, larger maximum stone diameter, lower eGFR, and modestly lower urinary calcium. *Escherichia coli* predominated among culture-positive women, whereas men showed a broader pathogen distribution. **Conclusions**: Positive admission urine culture was associated with greater stone burden and renal involvement in calcium oxalate stone disease, without a uniformly higher measured urinary biochemical profile. Culture status may provide clinically relevant phenotypic information alongside measured urinary biochemical assessment, although interpretation is limited by the absence of key CaOx-related urinary parameters such as oxalate, citrate, and supersaturation indices.

## 1. Introduction

Calcium oxalate (CaOx) stone formation is usually interpreted through the urinary physicochemical environment rather than through stone composition alone. The concentration of lithogenic ions and solutes, urine volume, and urine pH together influence CaOx supersaturation, crystal nucleation, crystal growth, aggregation, and retention [1]. In a prospective cohort analysis, Ferraro et al. [2] examined 9045 24-h urine collections from 6217 participants in the Health Professionals Follow-up Study and Nurses’ Health Studies I and II. Higher urinary calcium, phosphorus, and sodium were associated with a higher risk of incident symptomatic kidney stones, whereas higher urine volume and potassium were associated with a lower risk; urine pH was not significantly associated with stone formation. Although that study did not provide stone composition, it supports the broader principle that measured urinary biochemical and ionic parameters are clinically relevant to stone-forming biology. For CaOx stone formers, therefore, urinary calcium and other measured urinary solutes should be considered part of the biochemical background in which stones form, but they may not fully explain why crystals are retained and develop into clinically significant stones.

Sex is a major source of heterogeneity in both stone risk and urinary biochemical profiles. Ferraro et al. [3] analyzed 268,553 participants from the Health Professionals Follow-up Study and Nurses’ Health Studies, contributing 5,872,249 person-years of follow-up, and confirmed 10,302 incident kidney stones. The incidence rate was 271 per 100,000 person-years in men and 159 per 100,000 person-years in women, and men had a higher age-adjusted risk of stones. Importantly, men had higher urinary supersaturation for calcium oxalate and uric acid, which was attributed in part to differences in urinary oxalate, uric acid, phosphate, and urine pH. These data indicate that sex-related differences in stone disease are not limited to prevalence, but also involve the urinary environment in which CaOx crystallization occurs. Therefore, combining men and women in a single analysis may obscure clinically meaningful differences in urinary biochemical indicators and stone phenotype.

Infection has traditionally been linked to struvite stones caused by urease-producing organisms, but this framework does not fully account for the microbiological findings observed in non-struvite or CaOx-related stone disease [4,5]. In a prospective study of patients undergoing percutaneous nephrolithotomy, de Cógáin et al. [6]. identified a subgroup of patients with positive stone cultures despite the absence of struvite components, suggesting that non-struvite stone composition does not necessarily indicate absence of microbiological involvement. Under microaerobic conditions, *Escherichia coli* and *Proteus mirabilis* have been shown to promote calcium oxalate crystal growth and aggregation [7], and *E. coli* flagella have been implicated in CaOx crystallization, crystal growth, and aggregation [8]. Other bacterial products and epithelial responses, including *E. coli* outer membrane vesicle-associated factors, PPK1/flagellin-mediated oxidative injury, and crystal–cell adhesion pathways, have also been implicated in experimental CaOx models [9,10,11]. These observations provide a rationale for examining whether culture-positive CaOx stone formers differ in stone burden, stone location, pathogen distribution, and measured urinary biochemical profile.

Clinical urinary microbiome studies also suggest a bacteria–CaOx connection, but they do not answer the question addressed by the present study. Xie et al. [12] analyzed catheterized bladder urine from 22 men with calcium-based kidney stones and 21 age-matched healthy controls. Paired renal pelvic urine was also collected from stone patients. Although the study reported altered urinary microbiota in calcium-based stone formers, it included only men and involved a small mixed calcium-based stone cohort. In a later CaOx-focused study, Xie et al. [13] collected bladder urine from 68 adult CaOx stone patients and 54 age-matched healthy controls, and finally analyzed 48 CaOx stone formers and 52 controls after exclusions. The urinary microbiota structure differed between CaOx stone formers and controls, but patients with positive urine culture were excluded, and 24-h urinary lithogenesis-related factors were not evaluated. Taken together, previous studies have described urinary biochemical risk, sex differences in nephrolithiasis, non-struvite stone infection, experimental bacteria–crystal interactions, and urinary microbiota changes in CaOx stone formers. However, most of this evidence has come from mechanistic studies, microbiome-focused cohorts, or selected non-struvite infected stone populations. It remains unclear whether a routinely obtained admission urine culture identifies a clinically relevant profile among patients with FTIR-confirmed CaOx stones. The present study addresses this gap by integrating routine admission urine culture, FTIR-confirmed CaOx stone composition, sex-stratified clinical comparisons, biochemical testing variables, 24-h urinary parameters, and pathogen distribution among culture-positive patients.

In this retrospective study, we screened 2030 adults who underwent percutaneous nephrolithotomy or ureterorenoscopy and included 1257 patients with CaOx upper urinary tract stones and available admission urine culture results. We first compared men and women and then stratified the cohort by admission urine culture status within each sex. The primary objective was to evaluate whether positive admission urine culture was associated with stone burden, stone location, renal function-related indices, measured 24-h urinary biochemical parameters, and pathogen distribution in a sex-stratified CaOx cohort.

## 2. Materials and Methods

### 2.1. Patients and Study Design

We conducted a retrospective observational study of adult patients who underwent percutaneous nephrolithotomy or ureterorenoscopy for upper urinary tract stones at Beijing Tsinghua Changgung Hospital between January 2016 and December 2020.

Intraoperative stone specimens were collected and analyzed by Fourier transform infrared spectroscopy. Because most urinary stones contain mixed components and clinical classification is commonly based on the major stone component, patients were included if calcium oxalate monohydrate and/or calcium oxalate dihydrate accounted for ≥50% of the recorded stone composition [14]. Patients meeting this criterion are referred to as having calcium oxalate stones throughout this manuscript. Pure calcium oxalate stones were defined as stones composed entirely of calcium oxalate monohydrate and/or calcium oxalate dihydrate, whereas mixed calcium oxalate stones were defined as stones in which calcium oxalate monohydrate and/or calcium oxalate dihydrate accounted for ≥50% of the total composition but additional non-calcium oxalate components were also present [15].

**Figure 1:**
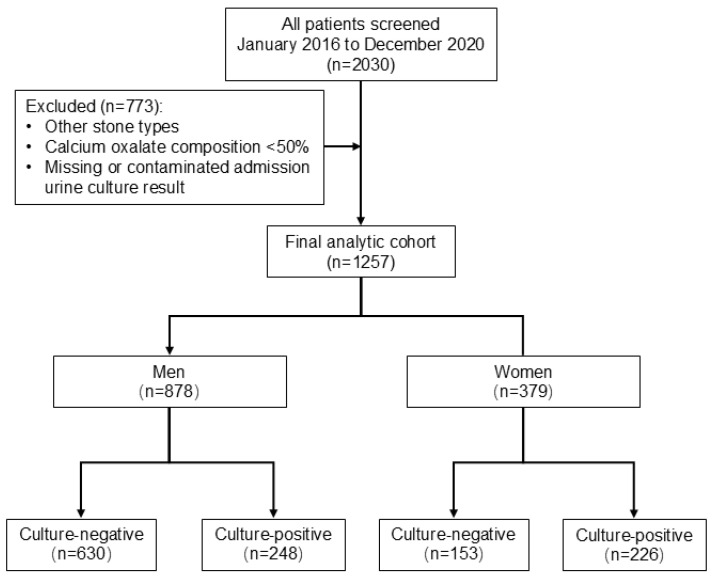
Patient selection flowchart.

A total of 2030 patients were screened. Patients were excluded if they had other stone types, calcium oxalate composition <50%, no admission urine culture result available, or contaminated admission urine culture. Patients with contaminated admission urine cultures were not classified as either culture-negative or culture-positive. The final study cohort comprised 1257 adults with calcium oxalate stones (Figure 1). For the primary analysis, the cohort was first compared by sex and was then stratified within each sex according to admission urine culture status.

The first midstream urine culture obtained at admission was used for analysis. At our institution, admission urine culture is routinely performed before inpatient antimicrobial treatment for patients scheduled for stone surgery and was therefore used to represent the bacteriological status at presentation. Repeated urine cultures were not required for inclusion; when additional cultures were available during hospitalization, only the first admission culture was used to define culture status. Information on antimicrobial exposure before hospital admission, including treatment received at outpatient clinics or other institutions, was not systematically available in the retrospective medical records and therefore could not be used for exclusion, stratification, or adjustment.

A culture was classified as positive when a predominant bacterial or fungal organism was identified at a colony count of ≥10⁵ CFU/mL, according to the conventional quantitative threshold for significant growth in midstream urine culture, and this threshold was applied uniformly to all reported organisms [16].

Cultures with no growth or colony counts below this threshold were classified as negative, unless the microbiology report indicated contamination. Samples reported as contaminated, including mixed growth without a predominant organism or laboratory contamination alerts, were excluded from analysis. No additional predefined exclusion was made solely on the basis of urease-producing organisms or radiologic features suggestive of classic infection stones, because eligibility was defined by FTIR-confirmed CaOx-predominant stone composition. The potential influence of additional non-CaOx components was evaluated separately by comparing pure and mixed CaOx stones and by including pure versus mixed CaOx composition in the adjusted model. For culture-positive patients, the reported organism was extracted from the microbiology report. For graphical presentation, less frequent urinary isolates were combined into the category “Other pathogens.”

Clinical variables collected for analysis included age, sex, body mass index (BMI), hypertension, diabetes mellitus, recurrent stone history, stone composition, stone laterality, stone location, maximum stone diameter, preoperative blood biochemical parameters, admission urine culture result, urine pH, and 24-h urinary biochemical measurements. Stone location was categorized as renal, ureteral, or renal and ureteral involvement. Maximum stone diameter was defined as the largest recorded stone diameter in the clinical record.

Twenty-four-hour urinary measurements were obtained from 24-h urine collections performed as part of routine preoperative metabolic evaluation. The recorded 24-h urinary variables included urine volume, calcium, uric acid, sodium, potassium, phosphorus, and chloride. These measurements were extracted from the hospital laboratory records. Because of the retrospective design, standardized documentation of collection completeness and urinary creatinine-based adequacy criteria was not consistently available; therefore, no additional exclusion was made on the basis of 24-h urinary creatinine excretion. Urinary oxalate, citrate, magnesium, and supersaturation indices were not routinely included in the hospital 24-h urinary panel during the study period and were not available in a consistent analyzable subset; therefore, no sensitivity analysis based on these parameters was performed.

### 2.2. Statistical Analysis

Continuous variables are presented as mean ± standard deviation, and categorical variables are presented as frequencies and percentages. Baseline comparisons were performed between men and women in the overall calcium oxalate cohort. Additional subgroup analyses compared culture-negative and culture-positive patients within each sex.

For continuous variables, between-group comparisons were performed using the independent-samples t test. Welch’s correction was applied when variance inequality was present. Categorical variables were compared using the chi-square test or Fisher’s exact test, as appropriate. All tests were two-sided, and *p* < 0.05 was considered statistically significant. For pathogen distribution, proportions were calculated among culture-positive patients within each sex, and exact binomial 95% confidence intervals were reported for each pathogen group. The overall pathogen distribution between women and men was compared using the chi-square test. Organism-specific sex differences were evaluated using Fisher’s exact tests and were interpreted descriptively because of multiple organism-level comparisons.

To assess the potential influence of additional non-CaOx components, pure and mixed CaOx stones were further compared. The variables compared included urine culture status, stone characteristics, biochemical testing variables, urine analysis variables, 24-h urinalysis variables, and pathogen distribution among culture-positive patients.

To account for potential confounding, multivariable logistic regression was performed with positive admission urine culture as the outcome. Covariates included sex, age, BMI, hypertension, diabetes mellitus, recurrent stone history, eGFR, pure versus mixed CaOx composition, maximum stone diameter, and stone location category. Age, eGFR, and maximum stone diameter were scaled per 10 years, per 10 mL/min/1.73 m², and per 10 mm, respectively. Results are reported as adjusted odds ratios (ORs) with 95% confidence intervals (CIs).

Additional adjusted logistic regression models were used to evaluate the association of positive urine culture with renal involvement and large stone burden. Renal involvement was defined as renal or renal-and-ureteral stone location versus ureteral location. Large stone burden was evaluated using maximum stone diameter thresholds of >20 mm and >30 mm. No imputation was performed for missing data. Descriptive comparisons were based on available observations for each variable, and multivariable logistic regression models were performed using complete cases for the variables included in each model. Given the number of variables compared across groups, analyses were organized by outcome domains. The primary outcome domain was the association between admission urine culture status and stone phenotype, including maximum stone diameter and renal involvement. Secondary outcome domains included renal function-related indices, measured 24-h urinary biochemical parameters, and pathogen distribution. Baseline demographic, biochemical, and urinary comparisons were used to characterize the cohort and were interpreted descriptively. No formal multiple-testing correction was applied; therefore, *p* values from secondary and descriptive comparisons were interpreted as exploratory.

Accordingly, the Results section first describes sex-related differences in the overall cohort, then compares culture-negative and culture-positive patients within each sex, and finally presents adjusted models for culture positivity and stone phenotype outcomes. Statistical analyses were performed using SPSS version 26.0 (IBM Corp., Armonk, NY, USA).

## 3. Results

### 3.1. Overall Comparison Between Men and Women with Calcium Oxalate Stones

A total of 1257 adults with calcium oxalate stones were included in the final study cohort, including 878 men (69.8%) and 379 women (30.2%) (Table 1). Women were older and had a slightly lower BMI than men. Diabetes mellitus was more frequent in women, whereas hypertension and recurrent stone history were similar between sexes.

Stone location and maximum stone diameter were comparable between men and women, although mixed CaOx stones and unilateral stones were slightly more frequent in women. Positive admission urine culture was substantially more frequent in women than in men (59.6% vs. 28.2%, *p* < 0.001). Compared with men, women had lower serum creatinine and serum uric acid, while eGFR and serum calcium were similar. In the 24-h urinary panel, women showed lower urinary calcium, uric acid, sodium, potassium, phosphorus, and chloride, whereas 24-h urine volume was similar.

### 3.2. Comparison Between Culture-Negative and Culture-Positive Men

Among men, 630 (71.8%) were culture-negative and 248 (28.2%) were culture-positive (Table 2). Culture-positive men were older and had a higher frequency of recurrent stone history than culture-negative men. Stone composition was similar between groups, but culture-positive men more often had renal stones and had a larger maximum stone diameter, indicating a greater stone burden in men with positive admission urine culture.

In the biochemical panel, culture-positive men had lower eGFR and lower serum calcium than culture-negative men. Other measured preoperative biochemical variables showed no clinically prominent differences. Across the 24-h urinary panel, urine pH, volume, calcium, uric acid, sodium, potassium, phosphorus, and chloride did not differ significantly between culture-negative and culture-positive men.

### 3.3. Comparison Between Culture-Negative and Culture-Positive Women

Among women, 153 (40.4%) were culture-negative and 226 (59.6%) were culture-positive (Table 3). Age and recurrent stone history were similar between groups, whereas BMI was slightly higher in culture-positive women. Stone composition did not differ by culture status. However, culture-positive women more often had renal stones and had a larger maximum stone diameter than culture-negative women, suggesting that culture status was more closely related to stone distribution and stone size than to stone composition.

Culture-positive women had higher preoperative creatinine and lower eGFR than culture-negative women, whereas serum uric acid, parathyroid hormone, and serum calcium were similar between groups. Within the 24-h urinary panel, urinary calcium was modestly lower in culture-positive women than in culture-negative women (1.81 ± 1.07 vs. 2.09 ± 1.19 mmol/D, *p* = 0.047), whereas urine pH, volume, uric acid, sodium, potassium, phosphorus, and chloride showed no significant differences. Overall, the differences associated with culture positivity in women were concentrated in stone distribution, stone size, and renal function-related indices, with only a modest difference in urinary calcium rather than a broad shift across the entire 24-h urinary profile.

### 3.4. Distribution of Urinary Pathogens in Culture-Positive Patients

**Figure 2:**
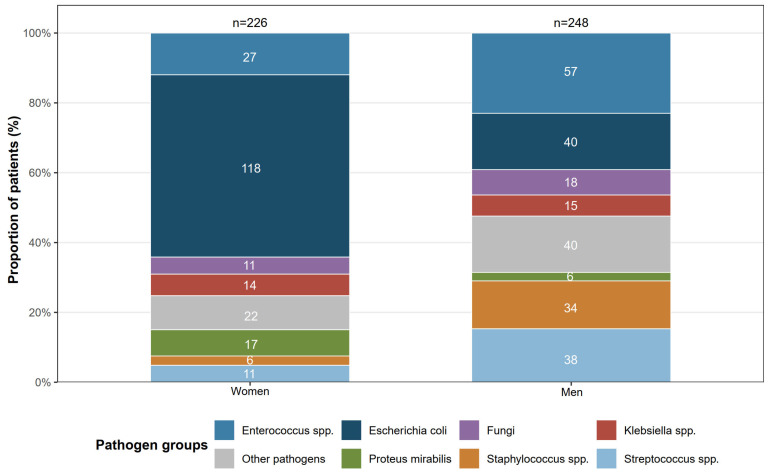
Urinary pathogen distribution among culture-positive women (*n* = 226) and men (*n* = 248). Stacked bars show within-sex proportions, and numbers inside bars indicate case counts. Less frequent isolates were grouped as “Other pathogens.” Exact percentages, 95% confidence intervals, and exploratory organism-specific *p* values are provided in Supplementary Table S3.

Among patients with positive urine culture, the overall pathogen distribution differed between women and men (*p* < 0.001; Figure 2 and Supplementary Table S3). In women, *Escherichia coli* was the predominant isolate, accounting for 118 of 226 culture-positive cases (52.2%, 95% CI 45.5–58.9), followed by *Enterococcus* spp. (27 cases, 11.9%, 95% CI 8.0–16.9). In men, the distribution was more heterogeneous. *Enterococcus* spp. were the most frequent isolate (57 of 248 cases, 23.0%, 95% CI 17.9–28.7), followed by *Escherichia coli* (40 cases, 16.1%, 95% CI 11.8–21.3), *Streptococcus* spp. (38 cases, 15.3%, 95% CI 11.1–20.4), and *Staphylococcus* spp. (34 cases, 13.7%, 95% CI 9.7–18.6). Organism-specific exploratory comparisons showed higher proportions of *Escherichia coli* and *Proteus mirabilis* in women, whereas *Enterococcus* spp., *Staphylococcus* spp., *Streptococcus* spp., and the heterogeneous “Other pathogens” category were more frequent in men. Overall, women showed a more concentrated pathogen profile dominated by *E. coli*, whereas men exhibited a broader distribution across several organism groups.

### 3.5. Pure Versus Mixed CaOx and Multivariable Analyses

To evaluate whether additional non-CaOx components influenced the relationship between stone composition and urine culture positivity, pure and mixed CaOx stones were compared. Positive admission urine culture was observed in 209 of 578 patients with pure CaOx stones (36.2%) and in 265 of 679 patients with mixed CaOx stones (39.0%), with no significant difference between groups (*p* = 0.296; Supplementary Table S1). Mixed CaOx stones were more frequently located in the kidney and had a slightly larger maximum stone diameter than pure CaOx stones. However, mixed composition was not independently associated with positive admission urine culture in the adjusted model (adjusted OR 1.00, 95% CI 0.77–1.31; *p* = 0.980; Table 4).

In the multivariable logistic regression model for positive admission urine culture, complete-case analysis included 1173 patients, including 435 with positive cultures. Women had higher adjusted odds of positive urine culture than men (adjusted OR 3.94, 95% CI 2.98–5.21; *p* < 0.001). Recurrent stone history was also associated with positive culture (adjusted OR 1.39, 95% CI 1.03–1.86; *p* = 0.029), as was larger maximum stone diameter (adjusted OR 1.12 per 10 mm, 95% CI 1.05–1.20; *p* < 0.001). Compared with renal stone location, ureteral stone location had lower adjusted odds of positive culture (adjusted OR 0.56, 95% CI 0.38–0.83; *p* = 0.004). By contrast, age, diabetes mellitus, and eGFR were not independently associated with positive culture in this adjusted model.

Additional adjusted models were used to evaluate stone phenotype outcomes (Supplementary Table S2). In the complete-case dataset used for these threshold-based models, 577 of 1173 patients (49.2%) had a maximum stone diameter >20 mm and 257 of 1173 patients (21.9%) had a maximum stone diameter >30 mm. Positive admission urine culture was associated with renal involvement after adjustment for age, sex, BMI, hypertension, diabetes mellitus, recurrent stone history, eGFR, and pure versus mixed CaOx composition (adjusted OR 2.23, 95% CI 1.55–3.21; *p* < 0.001). Positive culture was also associated with stone diameter >20 mm (adjusted OR 1.62, 95% CI 1.22–2.15; *p* < 0.001). However, when stone diameter >30 mm was used as a stricter threshold, the association was attenuated and did not reach statistical significance (adjusted OR 1.34, 95% CI 0.98–1.83; *p* = 0.067).

## 4. Discussion

In this retrospective surgical cohort of adults with CaOx upper urinary tract stones, sex and admission urine culture status were associated with distinct clinical patterns. Women had a substantially higher rate of positive admission urine culture than men, despite lower 24-h urinary calcium, uric acid, sodium, potassium, phosphorus, and chloride. Within both sexes, culture-positive patients tended to have larger stones and more frequent renal stone location, whereas the measured 24-h urinary profile did not show a uniformly higher lithogenic solute pattern. After multivariable adjustment, women, compared with men, had higher odds of positive admission urine culture; recurrent stone history, larger maximum stone diameter, and renal stone location also remained associated with positive admission urine culture. These findings suggest that culture-positive patients with CaOx stones were characterized primarily by differences in stone burden, renal involvement, and pathogen distribution, rather than by a simple increase in the measured urinary biochemical parameters. The phenotype-oriented adjusted models were consistent with this pattern. Positive admission urine culture was associated with renal involvement and with stone diameter >20 mm, whereas the association with stone diameter >30 mm was attenuated and did not reach statistical significance. Thus, the relationship between culture positivity and stone burden was supported by the continuous stone diameter analysis and the >20 mm threshold model, but was not statistically supported at the stricter >30 mm cutoff.

Previous cohort studies have established that urinary biochemical risk and stone incidence differ by sex [2,3]. Our study extends this concept in a CaOx surgical cohort by showing that sex differences in measured urinary solutes coexisted with major differences in urine culture positivity. Women in our cohort had lower urinary excretion of several measured solutes than men, but they had a substantially higher culture-positive rate. This pattern argues against a purely solute-centered interpretation of CaOx stone phenotype. Although urinary calcium and other solutes remain important components of the biochemical environment in which CaOx stones form, our data suggest that admission urine culture status may capture additional phenotypic information beyond the measured urinary solute profile, especially in women.

The most clinically relevant finding was that culture-positive patients had larger stones and more frequent renal stone location in both men and women. This pattern was not explained solely by stone composition. In the pure versus mixed CaOx analysis, positive urine culture rates were similar between pure and mixed CaOx stones, and mixed composition was not independently associated with positive admission urine culture after adjustment. Therefore, the association between culture positivity and stone phenotype was unlikely to be primarily attributable to the inclusion of mixed CaOx stones. However, because mixed urinary stones are compositionally heterogeneous, component-specific effects of non-CaOx minerals cannot be fully excluded. It was also not explained by a uniform increase in measured urinary biochemical parameters. In men, culture-positive status was associated with older age, recurrent stones, lower eGFR, renal stone location, and larger maximum stone diameter, but not with higher urinary calcium, uric acid, sodium, potassium, phosphorus, chloride, urine volume, or pH. In women, culture-positive status was associated with higher BMI, higher creatinine, lower eGFR, renal stone location, and larger stones, while urinary calcium was lower rather than higher. Therefore, the culture-positive CaOx phenotype in this cohort appeared to be concentrated in stone burden, renal involvement, and renal function-related indices rather than in a conventional high-solute urinary pattern.

This finding is consistent with the broader observation that bacteria can be present in non-struvite stones. Infection stones are classically associated with urease-producing organisms and struvite composition [4], but non-struvite stones cannot be assumed to be microbiologically irrelevant. In a prospective PCNL cohort, de Cógáin et al. [6]. showed that secondarily infected non-struvite stones accounted for approximately 20% of their surgical population and proposed several potential mechanisms linking bacteria with stone formation. Our cohort was restricted to CaOx stones, yet urine culture positivity was common and was associated with a heavier stone phenotype. These findings do not establish that bacteria initiated or accelerated CaOx stone formation; rather, they support the possibility that bacteriuria may coexist with, or mark, a distinct clinical expression of CaOx stone disease. Another important consideration is that midstream urine culture may underestimate or misclassify bacteria associated with the upper urinary tract or stone matrix. Mariappan et al. [17] reported that stone and renal pelvic urine cultures were better predictors of post-PCNL urosepsis than bladder urine culture. Paonessa et al. also showed discordance between preoperative bladder urine culture and intraoperative stone culture, indicating that voided or bladder urine is an imperfect surrogate for stone-associated microbiology [18]. Current EAU guidance recommends urine culture or urinary microscopy before planned stone treatment, treatment of urinary tract infection before stone removal, and collection of stone or renal pelvic urine culture during PCNL when possible [19]. In this context, the admission midstream urine culture used in our study should be interpreted as a clinically practical marker rather than a complete representation of renal pelvic or stone microbiology. A positive result is clinically relevant, but a negative result does not exclude bacteria within the stone or upper tract.

Experimental studies provide biological context for interpreting the association between culture positivity and stone phenotype. Barr-Beare et al. [20]. identified Enterobacteriaceae DNA and *E. coli* in CaOx stones. In their experimental model, *E. coli* aggregated around CaOx crystals. Mice exposed to both CaOx deposits and uropathogenic *E. coli* also showed increased renal CaOx deposits and innate immune activation. Chutipongtanate et al. [21]. demonstrated that intact viable bacteria, including *E. coli*, *Klebsiella pneumoniae*, *Staphylococcus aureus*, and *Streptococcus pneumoniae*, promoted CaOx crystal growth and aggregation in vitro. Amimanan et al. [9]. further reported that elongation factor Tu on *E. coli* outer membrane vesicles promoted CaOx crystal growth and aggregation, and Kanlaya et al. [8]. showed that *E. coli* flagella were involved in CaOx crystallization, crystal growth, and aggregation. Other experimental studies have linked *E. coli* exposure to oxidative injury, inflammatory signaling, and enhanced calcium oxalate monohydrate crystal–cell adhesion through epithelial pathways. These studies suggest that bacterial products and host epithelial responses can be linked to CaOx crystallization, aggregation, inflammation, and crystal–cell adhesion in experimental systems [10,11]. However, the present cohort did not directly assess bacteria–crystal interactions, epithelial injury, biofilm formation, or stone-associated microbiology. Therefore, the heavier stone phenotype observed in culture-positive patients should be interpreted as clinically associated with admission urine culture positivity, rather than as evidence of infection-mediated stone growth.

Clinical urinary microbiome studies also support a bacteria–CaOx connection, although most have not addressed routine culture-positive CaOx patients in the way examined here. Xie et al. [12]. reported altered urinary microbiota in a small cohort of men with calcium-based stones using catheterized bladder urine and paired renal pelvic urine. In a later CaOx-focused study, Xie et al. [13] found that urinary microbiota structure and functional pathways differed between CaOx stone formers and controls, but culture-positive patients were excluded and 24-h lithogenesis-related urinary factors were not evaluated. Kachroo et al. [22] used shotgun metagenomics and reported functional differences in the urinary tract microbiome of individuals with CaOx calculi, including reduced genes related to oxalate metabolism. Gao et al. [23] also reported distinct urinary microbial and metabolomic profiles in patients with nephrolithiasis compared with healthy controls. These studies suggest that urinary microbial ecology may be relevant to stone disease, but they do not clarify whether routine urine culture positivity identifies a distinct clinical phenotype among CaOx surgical patients. Our study adds a complementary clinical perspective by linking admission urine culture status with stone burden, stone location, renal function-related indices, and measured urinary biochemical parameters in a sex-stratified CaOx cohort.

The pathogen distribution in our study further supports the need for sex-specific interpretation. Among culture-positive women, *E. coli* was the predominant isolate, whereas culture-positive men showed a broader pathogen spectrum, with *Enterococcus* spp. as the most frequent group and substantial contributions from *E. coli*, *Streptococcus* spp., *Staphylococcus* spp., fungi, and other organisms. This distinction is important because much of the experimental evidence linking bacteria to CaOx crystallization, aggregation, epithelial injury, and crystal adhesion has focused on *E. coli* [8,9,10,11,20]. However, the broader pathogen distribution in men suggests that culture-positive CaOx stone disease should not be treated as a single microbiological entity. Reviews of the urinary microbiome in urolithiasis have proposed several pathways through which microbial communities may be linked to stone-related processes, including hyperoxaluria, CaOx supersaturation, biofilm formation, aggregation, and host inflammatory responses [24]. Therefore, both sex and organism distribution should be considered when interpreting culture-positive CaOx stone disease as a clinical phenotype, rather than as a single infection-driven entity.

Among women, culture-positive status was associated with a modestly lower 24-h urinary calcium level than culture-negative status (1.81 ± 1.07 vs. 2.09 ± 1.19 mmol/D; *p* = 0.047), although the large standard deviations indicate substantial overlap between groups. This finding occurred in the context of higher creatinine, lower eGFR, larger stone size, and more frequent renal involvement in culture-positive women. Differences in renal function may have influenced urinary solute excretion. In addition, dietary calcium and sodium intake before 24-h urine collection were not available, and medication history relevant to urinary calcium excretion, including thiazide use, was not consistently captured in the retrospective dataset. Given the borderline *p* value and broad dispersion of urinary calcium values, statistical variation or regression toward the mean may also have contributed to this observation. Therefore, the lower urinary calcium observed in culture-positive women should not be interpreted as evidence that lower calcium excretion protects against, or promotes, CaOx stone formation. Rather, it indicates that in this subgroup, larger stone burden and renal involvement were not accompanied by a broadly higher measured urinary solute profile. Because urinary oxalate, citrate, magnesium, and supersaturation indices were unavailable, the measured 24-h urinary panel captured only part of the CaOx-forming urinary environment in this subgroup.

Clinically, the association between admission urine culture positivity and larger renal stone burden suggests that culture status may provide relevant information even in patients with CaOx stones. Rather than defining an infection-driven CaOx subtype, a positive admission urine culture may serve as a readily available clinical marker of patients with greater renal involvement, larger stone burden, and distinct pathogen distribution. Guidelines already emphasize preoperative urine culture assessment and treatment of urinary tract infection before stone surgery [19]. In this context, our findings suggest that culture status may also carry phenotypic information in CaOx stone disease. At the same time, admission midstream urine culture does not establish the timing, source, or causal role of bacterial involvement. Together, culture status, stone burden, renal function, and measured urinary biochemical parameters describe complementary aspects of the CaOx stone phenotype. However, this phenotypic interpretation remains constrained by the absence of key CaOx-related urinary parameters, including oxalate, citrate, magnesium, and supersaturation indices. From a practical perspective, these findings suggest that culture-positive CaOx stone patients, particularly those with larger renal stone burden, may warrant closer perioperative infection assessment and surveillance. This does not imply that culture-positive CaOx stones should be managed as classic infection stones, but it supports careful review of admission urine culture results, attention to renal stone burden and renal function, and appropriate intraoperative sampling when clinically indicated [16]. Antibiotic use should remain guided by culture results, susceptibility patterns, clinical features, and institutional protocols, rather than by stone composition alone. Such an approach may help balance infection prevention with antibiotic stewardship, avoiding both under-recognition of clinically relevant bacteriuria and unnecessary broad or prolonged antimicrobial exposure.

This study has several strengths. First, the cohort was restricted to CaOx stones confirmed by Fourier transform infrared spectroscopy, reducing heterogeneity from other major stone types. Second, admission urine culture was obtained before inpatient antimicrobial treatment, allowing culture status to reflect the bacteriological condition at presentation. Third, the sample size was relatively large for a single-center surgical CaOx cohort. Fourth, the analysis was deliberately sex-stratified, which was important because men and women differed markedly in both urine culture positivity and measured urinary biochemical parameters. Finally, stone characteristics, renal function-related indices, urine pH, and measured 24-h urinary biochemical parameters were analyzed together, allowing culture positivity to be interpreted in relation to both stone phenotype and urinary biochemical background.

Several limitations should be acknowledged. First, the retrospective observational design precludes assessment of the temporal sequence between bacteriuria and stone growth. Therefore, the association between positive admission urine culture and heavier stone phenotype cannot establish the temporal sequence. Bacteriuria may have preceded stone enlargement, resulted from bacterial persistence within larger or more complex stones, or reflected secondary colonization of an established stone environment. Second, admission midstream urine culture was used rather than renal pelvic urine culture, stone culture, or sequencing. Midstream urine culture may not reliably reflect renal pelvic or stone-associated microbiology, and a negative admission culture does not exclude bacteria within the upper urinary tract or stone matrix. Third, the 24-h urinary measurements were obtained from routine clinical records, and standardized information on collection completeness was not consistently available. Urinary creatinine-based adequacy criteria were therefore not used to exclude potentially incomplete collections. In addition, the measured urinary biochemical panel was incomplete for CaOx pathophysiology because urinary oxalate, citrate, magnesium, and supersaturation indices were not routinely available in the hospital 24-h urinary panel during the study period. These parameters were not available in a consistent analyzable subset, precluding sensitivity analysis based on a more complete CaOx metabolic profile. Dietary intake before urine collection and medication use affecting urinary calcium excretion, such as thiazide use, were not consistently available, which may have influenced the interpretation of the urinary calcium findings. Fourth, multiple descriptive comparisons were performed without formal adjustment for multiplicity. Although the analysis was organized around primary and secondary outcome domains, isolated *p* values from secondary and descriptive comparisons should be interpreted cautiously. Fifth, information on antimicrobial exposure before hospital admission was not systematically available. Prior outpatient or external-institution antibiotic treatment may have reduced bacterial growth in admission midstream urine cultures and resulted in false-negative culture results, leading to underestimation of culture positivity. Therefore, admission urine culture status should be interpreted as a clinically available marker at presentation rather than as a complete measure of recent or partially treated urinary infection. Finally, no mechanistic assays were performed; therefore, the bacteria–crystal and bacteria–epithelium interpretations discussed above remain biologically plausible explanations rather than mechanisms directly demonstrated in this cohort.

In summary, admission urine culture positivity was associated with greater renal involvement, larger stone burden, and distinct pathogen distribution among patients with CaOx stones, but this pattern was not accompanied by a uniformly higher measured urinary biochemical profile. These findings support a clinically recognizable culture-positive CaOx phenotype rather than defining a metabolically complete or causally infection-driven subtype. This interpretation should be tempered by the absence of key CaOx-related urinary parameters, including urinary oxalate, citrate, magnesium, and supersaturation indices. Future studies should combine sex-stratified clinical analysis, complete 24-h urinary chemistry, renal pelvic or stone culture, microbiome sequencing, and mechanistic evaluation. Such studies are needed to clarify whether infection-related factors are involved in CaOx stone progression, accompany heavier stone phenotypes, or primarily reflect secondary colonization of established stones.

## Figures and Tables

**Table 1:** Baseline characteristics of men and women with calcium oxalate stones.

Characteristic	Men	Women	*p*-Value
Patients	878 (69.8%)	379 (30.2%)	
Age	49.5 ± 12.9	54.4 ± 12.5	<0.001
Body mass index	25.6 ± 4.0	24.9 ± 4.3	0.011
Hypertension			0.767
Yes	266 (30.3%)	118 (31.1%)	
No	612 (69.7%)	261 (68.9%)	
Diabetes			0.008
Yes	120 (13.7%)	74 (19.5%)	
No	758 (86.3%)	305 (80.5%)	
Recurrent stone			0.617
Yes	601 (68.5%)	254 (67.0%)	
No	277 (31.5%)	125 (33.0%)	
Biochemical testing			
Creatinine (μmol/L)	94.9 ± 65.5	74.9 ± 52.7	<0.001
Uric acid (μmol/L)	390.1 ± 89.4	313.6 ± 85.4	<0.001
eGFR (mL/min/1.73 m^2^)	89.7 ± 25.1	87.5 ± 26.3	0.173
Parathyroid hormone (pg/mL)	55.3 ± 51.2	67.7 ± 102.5	0.049
Serum calcium (mmol/L)	2.26 ± 0.14	2.26 ± 0.17	0.467
Stone characteristics			
Stone type			0.045
Pure CaOx	420 (47.8%)	158 (41.7%)	
Mixed CaOx	458 (52.2%)	221 (58.3%)	
Laterality of stones			0.022
Unilateral	654 (74.5%)	305 (80.5%)	
Bilateral	224 (25.5%)	74 (19.5%)	
Location of stones			0.803
Renal	603 (68.7%)	264 (69.7%)	
Ureteral	164 (18.7%)	65 (17.2%)	
Renal and ureteral	111 (12.6%)	50 (13.2%)	
Maximum stone diameter (mm)	25.6 ± 19.7	25.6 ± 19.2	0.978
Urine Analysis			
Urine culture			<0.001
Positive	248 (28.2%)	226 (59.6%)	
Negative	630 (71.8%)	153 (40.4%)	
Urine pH	6.22 ± 0.58	6.32 ± 0.47	0.120
24-h urinalysis			
Volume (mL/D)	2024.3 ± 742.4	2078.6 ± 807.3	0.338
Calcium (mmol/D)	2.30 ± 1.27	1.91 ± 1.12	<0.001
Uric acid (μmol/D)	1674.8 ± 768.6	1320.0 ± 620.4	<0.001
Sodium (mmol/D)	89.7 ± 37.1	73.4 ± 35.1	<0.001
Potassium (mmol/D)	17.71 ± 8.01	15.72 ± 7.71	<0.001
Phosphorus (mmol/D)	9.90 ± 4.98	7.26 ± 6.13	<0.001
Chloride (mmol/D)	72.4 ± 32.1	59.5 ± 29.9	<0.001

**Table 2:** Characteristics of culture-negative and culture-positive men with calcium oxalate stones.

Characteristic	Culture-Negative	Culture-Positive	*p*-Value
Patients	630 (71.8%)	248 (28.2%)	
Age	48.5 ± 12.5	52.0 ± 13.4	<0.001
Body mass index	25.5 ± 3.7	25.8 ± 4.7	0.409
Recurrent stone			<0.001
Yes	408 (64.8%)	193 (77.8%)	
No	222 (35.2%)	55 (22.2%)	
Biochemical testing			
Creatinine (μmol/L)	92.0 ± 58.0	102.1 ± 81.2	0.075
Uric acid (μmol/L)	392.1 ± 84.6	385.1 ± 100.1	0.344
eGFR (mL/min/1.73 m^2^)	91.1 ± 23.7	86.0 ± 28.0	0.011
Parathyroid hormone (pg/mL)	54.4 ± 44.5	57.7 ± 65.6	0.519
Serum calcium (mmol/L)	2.27 ± 0.13	2.23 ± 0.15	0.003
Stone characteristics			
Stone type			0.806
Pure CaOx	303 (48.1%)	117 (47.2%)	
Mixed CaOx	327 (51.9%)	131 (52.8%)	
Location of stones			<0.001
Renal	408 (64.8%)	195 (78.6%)	
Ureteral	140 (22.2%)	24 (9.7%)	
Renal and ureteral	82 (13.0%)	29 (11.7%)	
Maximum stone diameter (mm)	23.6 ± 16.5	30.7 ± 25.8	<0.001
Urine Analysis			
Urine pH	6.19 ± 0.60	6.29 ± 0.50	0.380
24-h urinalysis			
Volume (mL/D)	2002.4 ± 724.8	2072.0 ± 779.5	0.296
Calcium (mmol/D)	2.32 ± 1.25	2.28 ± 1.32	0.724
Uric acid (μmol/D)	1695.3 ± 778.0	1629.4 ± 747.6	0.342
Sodium (mmol/D)	90.7 ± 38.1	87.4 ± 34.6	0.328
Potassium (mmol/D)	17.97 ± 7.92	17.16 ± 8.18	0.262
Phosphorus (mmol/D)	9.98 ± 4.79	9.72 ± 5.38	0.566
Chloride (mmol/D)	73.0 ± 32.7	71.0 ± 30.7	0.482

**Table 3:** Characteristics of culture-negative and culture-positive women with calcium oxalate stones.

Characteristic	Culture-Negative	Culture-Positive	*p*-Value
Patients	153 (40.4%)	226 (59.6%)	
Age	54.9 ± 12.1	54.1 ± 12.8	0.552
Body mass index	24.4 ± 3.6	25.3 ± 4.7	0.040
Recurrent stone			0.312
Yes	98 (64.1%)	156 (69.0%)	
No	55 (35.9%)	70 (31.0%)	
Biochemical testing			
Creatinine (μmol/L)	68.2 ± 43.3	79.5 ± 57.8	0.031
Uric acid (μmol/L)	311.0 ± 100.7	315.3 ± 73.6	0.672
eGFR (mL/min/1.73 m^2^)	91.5 ± 23.8	84.9 ± 27.6	0.017
Parathyroid hormone (pg/mL)	67.9 ± 97.5	67.5 ± 106.0	0.976
Serum calcium (mmol/L)	2.27 ± 0.18	2.26 ± 0.16	0.374
Stone characteristics			
Stone type			0.638
Pure CaOx	66 (43.1%)	92 (40.7%)	
Mixed CaOx	87 (56.9%)	134 (59.3%)	
Location of stones			0.022
Renal	100 (65.4%)	164 (72.6%)	
Ureteral	36 (23.5%)	29 (12.8%)	
Renal and ureteral	17 (11.1%)	33 (14.6%)	
Maximum stone diameter (mm)	22.6 ± 19.0	27.7 ± 19.1	0.014
Urine Analysis			
Urine pH	6.21 ± 0.46	6.39 ± 0.47	0.096
24-h urinalysis			
Volume (mL/D)	1987.2 ± 715.4	2132.6 ± 854.4	0.158
Calcium (mmol/D)	2.09 ± 1.19	1.81 ± 1.07	0.047
Uric acid (μmol/D)	1360.5 ± 696.2	1296.0 ± 571.8	0.441
Sodium (mmol/D)	73.7 ± 35.8	73.2 ± 34.7	0.912
Potassium (mmol/D)	15.84 ± 8.31	15.65 ± 7.35	0.844
Phosphorus (mmol/D)	8.19 ± 8.79	6.69 ± 3.62	0.112
Chloride (mmol/D)	60.3 ± 30.4	59.1 ± 29.7	0.755

**Table 4:** Multivariable logistic regression analysis of factors associated with positive admission urine culture.

Characteristic	Adjusted OR	95% CI	*p*-Value
Female sex vs. male sex	3.94	2.98–5.21	<0.001
Age, per 10 years	1.08	0.96–1.21	0.202
BMI, per 1 kg/m^2^	1.03	1.00–1.07	0.035
Hypertension, yes vs. no	1.11	0.83–1.49	0.489
Diabetes, yes vs. no	0.99	0.68–1.44	0.946
Recurrent stone, yes vs. no	1.39	1.03–1.86	0.029
eGFR, per 10 mL/min/1.73 m^2^	0.95	0.90–1.00	0.065
Mixed vs. pure CaOx	1.00	0.77–1.31	0.980
Maximum stone diameter, per 10 mm	1.12	1.05–1.20	<0.001
Ureteral vs. renal	0.56	0.38–0.83	0.004
Renal and ureteral vs. renal	0.92	0.62–1.35	0.665

**Notes:** BMI, body mass index; eGFR, estimated glomerular filtration rate; CaOx, calcium oxalate.

## Data Availability

The data presented in this study are available on reasonable request from the corresponding author. The data are not publicly available because they contain patient-level clinical information from a retrospective clinical cohort and are subject to institutional privacy and ethical restrictions.

## Supplementary Materials

The following supporting information can be downloaded at: https://www.mdpi.com/article/10.3390/pathogens15070692/s1, Table S1: Comparison between pure and mixed calcium oxalate stones; Table S2: Exploratory adjusted models for stone phenotype; Table S3: Pathogen distribution among culture-positive patients by sex.
