# Supplementary material for: Sex Differences in Admission Urine Culture Positivity, Pathogen Distribution, and Clinical Characteristics Among Patients with Calcium Oxalate Stones
Source: Pathogens. 2026 Jun 30;15(7):692. doi: 10.3390/pathogens15070692 (PMC13414899; doi:10.3390/pathogens15070692)
Supplement: Supplementary file 1 [file pathogens-15-00692-s001.zip › pathogens-4337977-supplementary.pdf]

Supplementary Table S1. Comparison between pure and mixed calcium oxalate stones

Characteristic	Pure CaOx	Mixed CaOx	P-value
Urine culture			0.296
Negative	369 (63.8%)	414 (61.0%)	
Positive	209 (36.2%)	265 (39.0%)	
Stone location			0.002
Renal	371 (64.2%)	496 (73.0%)	
Ureteral	127 (22.0%)	102 (15.0%)	
Renal and ureteral	80 (13.8%)	81 (11.9%)	
Maximum stone diameter, mm	24.2 ± 17.7	26.7 ± 21.0	0.023
Biochemical testing			
Creatinine (μmol/L)	92.7 ± 71.6	85.6 ± 53.5	0.051
Uric acid (μmol/L)	375.1 ± 93.2	360.9 ± 95.9	0.012
eGFR (mL/min/1.73 m²)	86.1 ± 25.5	91.5 ± 25.1	<0.001
Parathyroid hormone (pg/mL)	58.1 ± 75.5	59.8 ± 66.0	0.693
Serum calcium (mmol/L)	2.26 ± 0.13	2.26 ± 0.16	0.554
Urine Analysis			
Urine pH	6.54 ± 0.72	6.24 ± 0.53	0.062
Volume (mL/D)	2,024.3 ± 786.0	2,055.7 ± 744.0	0.552
Calcium (mmol/D)	2.14 ± 1.29	2.22 ± 1.20	0.361
Uric acid (μmol/D)	1,582.1 ± 754.2	1,548.3 ± 734.9	0.513
Sodium (mmol/D)	84.7 ± 36.9	84.4 ± 37.5	0.921
Potassium (mmol/D)	17.01 ± 7.81	17.16 ± 8.11	0.784
Phosphorus (mmol/D)	9.19 ± 4.95	8.98 ± 5.93	0.569
Chloride (mmol/D)	68.8 ± 31.4	68.0 ± 32.5	0.733
Pathogen distribution among culture-positive patients			0.180
Enterococcus spp.	34 (16.3%)	50 (18.9%)	
Escherichia coli	69 (33.0%)	89 (33.6%)	
Fungi	18 (8.6%)	11 (4.2%)	
Klebsiella spp.	9 (4.3%)	20 (7.5%)	
Other pathogens	32 (15.3%)	30 (11.3%)	
Proteus mirabilis	11 (5.3%)	12 (4.5%)	
Staphylococcus spp.	13 (6.2%)	27 (10.2%)	
Streptococcus spp.	23 (11.0%)	26 (9.8%)	

Notes: CaOx, calcium oxalate; eGFR, estimated glomerular filtration rate.

Supplementary Table S2. Exploratory adjusted models for stone phenotype

Model	Outcome	Variable	Adjusted OR	95% CI	P-value
Model 1	Renal involvement	Positive urine culture, yes vs. no	2.23	1.55–3.21	<0.001
		Age, per 10 years	0.94	0.81–1.07	0.345
		Female sex vs. male sex	0.88	0.62–1.27	0.499
		BMI, per 1 kg/m²	0.96	0.93–1.00	0.041
		Hypertension, yes vs. no	1.00	0.70–1.41	0.979
		Diabetes, yes vs. no	0.95	0.62–1.46	0.827
		Recurrent stone, yes vs. no	3.17	2.34–4.30	<0.001
		eGFR, per 10 mL/min/1.73 m²	0.96	0.89–1.03	0.216
		Mixed vs. pure CaOx	1.42	1.04–1.93	0.026
Model 2A	Stone diameter >20 mm	Positive urine culture, yes vs. no	1.62	1.22–2.15	<0.001
		Age, per 10 years	1.04	0.92–1.17	0.524
		Female sex vs. male sex	0.82	0.60–1.11	0.192
		BMI, per 1 kg/m²	1.02	0.99–1.06	0.156
		Hypertension, yes vs. no	0.97	0.71–1.31	0.830
		Diabetes, yes vs. no	0.74	0.51–1.09	0.132
		Recurrent stone, yes vs. no	1.91	1.43–2.55	<0.001
		eGFR, per 10 mL/min/1.73 m²	0.91	0.86–0.96	0.001
		Ureteral vs. renal	0.05	0.03–0.09	<0.001
		Renal and ureteral vs. renal	0.50	0.34–0.71	<0.001
		Mixed vs. pure CaOx	0.94	0.72–1.23	0.650
Model 2B	Stone diameter >30 mm	Positive urine culture, yes vs. no	1.34	0.98–1.83	0.067
		Age, per 10 years	1.04	0.91–1.20	0.548
		Female sex vs. male sex	0.96	0.69–1.35	0.829
		BMI, per 1 kg/m²	1.02	0.98–1.06	0.309
		Hypertension, yes vs. no	1.13	0.81–1.58	0.478
		Diabetes, yes vs. no	0.85	0.54–1.33	0.466
		Recurrent stone, yes vs. no	2.20	1.51–3.22	<0.001
		eGFR, per 10 mL/min/1.73 m²	0.90	0.85–0.95	<0.001
		Ureteral vs. renal	0.04	0.01–0.14	<0.001
		Renal and ureteral vs. renal	0.31	0.19–0.53	<0.001
		Mixed vs. pure CaOx	1.00	0.74–1.35	0.992

Notes: Model 1 evaluated renal involvement, defined as renal or renal-and-ureteral stone location versus ureteral-only location. Models 2A and 2B evaluated maximum stone diameter thresholds of >20 mm and >30 mm, respectively. Adjusted covariates are described in Methods. OR, odds ratio; CI, confidence interval; BMI, body mass index; eGFR, estimated glomerular filtration rate; CaOx, calcium oxalate.

Supplementary Table S3. Pathogen distribution among culture-positive patients by sex

Pathogen group	Women, *n*=226	95% CI	Men, *n*=248	95% CI	P-value
Enterococcus spp.	27 (11.9%)	8.0–16.9	57 (23.0%)	17.9–28.7	0.002
Escherichia coli	118 (52.2%)	45.5–58.9	40 (16.1%)	11.8–21.3	<0.001
Fungi	11 (4.9%)	2.5–8.5	18 (7.3%)	4.4–11.2	0.339
Klebsiella spp.	14 (6.2%)	3.4–10.2	15 (6.0%)	3.4–9.8	1.000
Other pathogens	22 (9.7%)	6.2–14.4	40 (16.1%)	11.8–21.3	0.042
Proteus mirabilis	17 (7.5%)	4.4–11.8	6 (2.4%)	0.9–5.2	0.010
Staphylococcus spp.	6 (2.7%)	1.0–5.7	34 (13.7%)	9.7–18.6	<0.001
Streptococcus spp.	11 (4.9%)	2.5–8.5	38 (15.3%)	11.1–20.4	<0.001

Notes: CI, confidence interval. Organism-specific P values were interpreted descriptively.
